# Comparison of the ABC and ACMG systems for variant classification

**DOI:** 10.1038/s41431-024-01617-8

**Published:** 2024-05-22

**Authors:** Gunnar Houge, Eirik Bratland, Ingvild Aukrust, Kristian Tveten, Gabrielė Žukauskaitė, Ivona Sansovic, Alejandro J. Brea-Fernández, Karin Mayer, Teija Paakkola, Caoimhe McKenna, William Wright, Milica Keckarevic Markovic, Dorte L. Lildballe, Michal Konecny, Thomas Smol, Pia Alhopuro, Estelle Arnaud Gouttenoire, Katharina Obeid, Albena Todorova, Milena Jankovic, Joanna M. Lubieniecka, Maja Stojiljkovic, Marie-Pierre Buisine, Bjørn Ivar Haukanes, Marie Lorans, Hanno Roomere, François M. Petit, Maria K. Haanpää, Claire Beneteau, Belén Pérez, Dijana Plaseska-Karanfilska, Matthias Rath, Nico Fuhrmann, Bibiana I. Ferreira, Coralea Stephanou, Wenche Sjursen, Aleš Maver, Cécile Rouzier, Adela Chirita-Emandi, João Gonçalves, Wei Cheng David Kuek, Martin Broly, Lonneke Haer-Wigman, Meow-Keong Thong, Sok-Kun Tae, Michaela Hyblova, Johan T. den Dunnen, Andreas Laner

**Affiliations:** 1 Department of Medical Genetics, Haukeland University Hospital, Bergen, Norway; 2 Department of Medical Genetics, Telemark Hospital Trust, Skien, Norway; 3 Department of Human and Medical Genetics, Institute of Biomedical Sciences, Faculty of Medicine, Vilnius University, Vilnius, Lithuania; 4 Department of Medical and Laboratory Genetics, Endocrinology and Diabetology, Childrens’ Hospital Zagreb, University of Zagreb School of Medicine, Zagreb, Croatia; 5 Grupo de Medicina Xenómica, Universidade de Santiago de Compostela, CIBERER), Santiago de Compostela, Spain; 6 Center for Human Genetics and Laboratory Diagnostics, MVZ Martinsried GmbH, Martinsried, Germany; 7 Nordlab Wellbeing Service Group, Genetics Laboratory, Oulu, Finland; 8 Northern Ireland Regional Molecular Diagnostic Service, Belfast, Northern Ireland; 9 Center for Applied and Forensic Molecular Genetics, Faculty of Biology, University of Belgrade, Belgrade, Serbia; 10 Department of Molecular Medicine, Aarhus University Hospital, Aarhus, Denmark; 11 Laboratory of Genomic Medicine, GHC GENETICS SK, Bratislava, Slovakia; 12 Department of Biology, Institute of Biology and Biotechnology, Faculty of Natural Sciences, University of ss. Cyril and Methodius in Trnava, Trnava, Slovakia; 13 Institut de Genetique Medicale—CHU Lille, Lille, France; 14 HUS Diagnostic Center, Laboratory of Genetics, University of Helsinki and Helsinki University Hospital, Helsinki, Finland; 15 MEDISYN Genetics, Chemin d’Entre-Bois 21, Lausanne, Switzerland; 16 Molecular Diagnostics, Institute of Human Genetics, University Hospital Heidelberg, Heidelberg, Germany; 17 Genetic Medico-Diagnostic Laboratory “Genica” and Genome Center Bulgaria, Sofia, Bulgaria; 18 Neurology Clinic UCCS, Faculty of Medicine, University of Belgrade, Belgrade, Serbia; 19 Humangenetik, Ruhr-Universität Bochum, Bochum, Germany; 20 Institute of Molecular Genetics and Genetic Engineering, University of Belgrade, Belgrade, Serbia; 21 Molecular Oncogenetics, Department of Biochemistry and Molecular Biology, Lille University Hospital, Lille, France; 22 Univ. Lille, CNRS, Inserm, CHU Lille, UMR9020-U1277, CANTHER, Lille, France; 23 Department of Clinical Genetics, Aarhus University Hospital, Aarhus, Denmark; 24 Department of laboratory genetics, Genetics and Personalized Medicine Clinic, Tartu University Hospital, Tartu, Estonia; 25 Department of Oncopharmacology, Centre Antoine Lacassagne, Nice, France; 26 Department of Genomics, Turku University Hospital, Turku, Finland; 27 CHU Bordeaux, Service de Génétique Médicale, F-33000 Bordeaux, France; 28 Genetics Department of CEDEM, Universidad Autónoma de Madrid, Madrid, Spain; 29 Research Centre for Genetic Engineering and Biotechnology “Georgi D. Efremov”, Macedonian Academy of Sciences and Arts, Skopje, North Macedonia; 30 Institute for Molecular Medicine, MSH Medical School Hamburg, Hamburg, Germany; 31 Department of Human Genetics, University Medicine Greifswald and Interfaculty Institute of Genetics and Functional Genomics, University of Greifswald, Greifswald, Germany; 32 Institute of Human Genetics, University of Cologne, Cologne, Germany; 33 GENELAB by ABC, Faro, Portugal; 34 Faculty of Medicine and Biomedical Sciences, University of Algarve, Campus de Gambelas, Faro, Portugal; 35 Molecular Genetics Thalassemia Department, The Cyprus Institute of Neurology and Genetics, Nicosia, Cyprus; 36 Department of Medical Genetics, St Olavs Hospital, Trondheim, Norway; 37 Clinical Institute of Genomic Medicine, Ljubljana, Slovenia; 38 Department of Medical Genetics, National Centre for Mitocondrial Diseases, CHU de NICE, Université Côte d’Azur, Nice, France; 39 CNRS, INSERM, IRCAN, Université Côte d’Azur, Nice, France; 40 Department of Microscopic Morphology Genetics Discipline, Center of Genomic Medicine, “Victor Babes” University of Medicine and Pharmacy Timisoara, Timisoara, Romania; 41 Human Genetics Department, National Institute of Health Dr Ricardo Jorge, Lisbon, Portugal; 42 Molecular Diagnosis Centre, Department of Laboratory Medicine, National University Hospital, Kent Ridge, Singapore; 43 Laboratory of Rare and Autoinflammatory Genetic Diseases, Department of Genetics-LBM, Montpellier University Hospital, Montpellier, France; 44 Department of Human Genetics, Radboud University Medical Center, Nijmegen, The Netherlands; 45 Genetics and Metabolism Unit, Department of Pediatrics, Faculty of Medicine, University of Malaya, Kuala Lumpur, Malaysia; 46 Department of Genetics, Medirex, Bratislava, Slovakia; 47 Department of Human Genetics, Leiden University Medical Center, Leiden, The Netherlands; 48 Medizinisch Genetisches Zentrum (MGZ) München, Munich, Germany

**Keywords:** Genetic testing, Genetic predisposition to disease, Genetic testing

## Abstract

The ABC and ACMG variant classification systems were compared by asking mainly European clinical laboratories to classify variants in 10 challenging cases using both systems, and to state if the variant in question would be reported as a relevant result or not as a measure of clinical utility. In contrast to the ABC system, the ACMG system was not made to guide variant reporting but to determine the likelihood of pathogenicity. Nevertheless, this comparison is justified since the ACMG class determines variant reporting in many laboratories. Forty-three laboratories participated in the survey. In seven cases, the classification system used did not influence the reporting likelihood when variants labeled as “maybe report” after ACMG-based classification were included. In three cases of population frequent but disease-associated variants, there was a difference in favor of reporting after ABC classification. A possible reason is that ABC step C (standard variant comments) allows a variant to be reported in one clinical setting but not another, e.g., based on Bayesian-based likelihood calculation of clinical relevance. Finally, the selection of ACMG criteria was compared between 36 laboratories. When excluding criteria used by less than four laboratories (<10%), the average concordance rate was 46%. Taken together, ABC-based classification is more clear-cut than ACMG-based classification since molecular and clinical information is handled separately, and variant reporting can be adapted to the clinical question and phenotype. Furthermore, variants do not get a clinically inappropriate label, like pathogenic when not pathogenic in a clinical context, or variant of unknown significance when the significance is known.

## Introduction

For the last 10 years, the prevailing system for classification of genetic variants has been the ACMG-AMP system, developed by the American College of Medical Genetics and Genomics and the Association for Molecular Pathology, and further developed by ClinGen´s Sequence Variant Interpretation Working Groups [1–3]. The ACMG-AMP system was made to determine a variant’s likelihood of pathogenicity, and it has a defined list of classification criteria. It is especially well suited for dominant variants of moderate-to-high penetrance. The system has also been adapted to copy number variations and variants in mitochondrial DNA [4, 5]. Currently the ACMG-AMP system is in the process of further development, including setting up a framework for classification of low-penetrant variants and risk alleles [6], and to make classification more point-based [7].

More recently, an alternative system was developed by a European Society of Human Genetics’ task force and called the ABC system [8, 9]. This system was made to guide and standardize variant reporting, and to answer the question of variant relevance in a given clinical setting. Unlike the ACMG-AMP system, the ABC system can classify any type of genetic or genomic variation, including variants of no medical relevance, with no preference for certain variant types. One reason is that the ABC system separates functional grading of a variant (ABC step A) from clinical grading of a variant’s relevance (ABC step B). In ABC step C, a standardized variant comment can be selected. Comment selection can be adapted to the clinical question and the patient’s phenotype.

Here we have conducted a survey comparing ACMG- and ABC-based variant classification. We have also investigated if the classification systems influenced the likelihood of a variant being reported, and how consistent the selection of ACMG criteria was between laboratories.

## Methods

A questionnaire (Supplementary File S1) starting with an invitation letter, a description of the ACMG and ABC systems, and a list of ten challenging real-world cases (variants and associated clinical data) selected from the first author’s clinical practice, was sent to various laboratories in Europe based on personal contacts or via the European Society of Human Genetics’ link to European National Human Genetics Societies. Late invites were also sent to laboratories in Asia, Africa and the US, only two participated, bringing the number of participating laboratories to 43. All cases were stripped of information that hypothetically could lead to identification of the subjects or families. The respondents were asked to grade variants according to both ACMG and ABC systems, and to select a standardized comment in ABC step C. This step determines if a variant will be reported or not. We also asked participants to state if the variant would have been reported after ACMG classification only. Even though the ACMG system was not made to guide variant reporting, many laboratories use it for this purpose. Some laboratories answered “maybe” to this latter question (Table 1). It was optional to list the selection of ACMG criteria, and 33–36 laboratories did this, depending on the case (Table 2). Finally, laboratories were asked if they found ABC step C (standardized variant comments) useful, since this is a discriminating feature of the ABC system. Laboratories were also invited to give general comments about ABC classification, and these have been used to improve the system. Questionnaires were returned by e-mail, and responses were plotted in Excel, including all the comments (Supplementary File S2). In a separate Excel sheet, the choices of ACMG criteria were plotted, one sheet per variant (Supplementary File S3).

**Table 1:** Result of variant classification: average grading and percentage of variant reporting.

Case #	Gene	Variant^a^	Clinical fit with variant	ACMG^b^ grade	ABC-A^c^ grade	ABC-B^c^ grade	% reported after ACMG Incl maybe	% reported after ACMG excl maybe	% reported after ABC
1	*CHEK2*	p.(Ile200Thr)	Good	3,5 - VUS	4,3 - LFE	2,3 - RF	83	71	91
2	*CACNA1A*	p.(Arg1437Gln)	Good	3,2 - VUS	3,1 - HFE	1,9 - RF	95	81	95
3	*ADAMTS18*	p.(Arg246Ter) p.(Arg573Pro)	Excellent	4,3 - LP 3,3 - VUS	4,4 - LFE 3,1 - HFE	3,1 - P 2,0 - RF	95	95	100
4	*HUWE1*	p.(Glu4315Lys)	Good	3,3 - VUS	3,4 - HFE	1,9- RF	95	74	100
5	*COL5A1*	Thr915Met	Good	2,9 - VUS	2,6 - HFE	1,5 - VOI	80	54	79
6	Deletion of *ANK2*	1 Mb de novo deletion	? Normal fetus	3,8 - LP	3,6 - LFE	0,8 - VOI	67	57	65
7	Duplication of 16p11.2	227 kb duplication	Moderate	3,1 - VUS	3,0 - HFE	1,5 - VOI	71	60	79
8	*PTPN11*	p.(Gly268Ser)	Poor	4,2 - LP	3,9 - LFE	1,2 - VOI	64	50	67
9	*TNFRSF1A*	p.(Arg121Gln)	Moderate	2,6 - VUS	3,1 - HFE	2,0 - RF	64	40	81
10	*ABCA4*	p.(Asn1868Ile)	Excellent	2,7 - VUS	3,6 - LFE	1,8 - RF	67	50	86

*LP* likely pathogenic, *VUS* variant of unknown significance, *LFE* likely functional effect, *HFE* hypothetical functional effect, *RF* risk factor, *VOI* variant of interest, *P* pathogenic.

^a^For variant details, see Supplementary File S1.

^b^The ACMG grades are the average of the grades given by all participating laboratories, see Supplementary File S2 for details. Please note that to be able to calculate this, ACMG classes were converted to numbers: P = 5, LP = 4, VUS = 3, LB = 2 and B = 1.

^c^ABC grades are the average of ABC-A and ABC-B grading, see Supplementary File S2 for details.

**Table 2:** ACMG criteria used by 33–36 of the 43 laboratories.

Case #	Gene	Variant	Clinical fit	ACMG concordance in criteria selection (%)^a^	Number of ACMG criteria used in >50% of laboratories
1	*CHEK2*	p.(Ile200Thr)^b^	Good	32	3
2	*CACNA1A*	p.(Arg1437Gln)	Good	44	2
3	*ADAMTS18*	p.(Arg246Ter) p.(Arg573Pro)	Excellent	72 50	2 2
4	*HUWE1*	p.(Glu4315Lys)	Good	50	3
5	*COL5A1*	p.(Thr915Met)	Good	37	1
8	*PTPN11*	p.(Gly268Ser)	Poor	63	4
9	*TNFRSF1A*	p.(Arg121Gln)^b^	Moderate	37	2
10	*ABCA4*	p.(Asn1868Ile)^b^	Excellent	27	0

^a^Criteria selected by less than 10% of the laboratories (3 or less) were excluded from the calculation.

^b^Known low-penetrant variant/hypomorphic allele.

Concordance rates were calculated as number of laboratories that selected a given criterium divided by the number of laboratories that responded, see Supplementary File 3 for details and calculations.

## Results

Comparing ABC step A to the five ACMG variant classes (B-benign, LB-likely benign, VUS-variant of unknown significance, LP-likely pathogenic, P-pathogenic, in this case numbered as 1–5 to be able to compare averages), the results of variant grading were quite similar with two exceptions (Table 1; case 1 and 10, see also Supplementary File 2 for statistical comparisons). An ACMG VUS corresponds in these cases to ABC-A grade HFE (variant of hypothetical functional effect), and an ACMG LP corresponds to ABC-A grade LFE (variant of likely functional effect). The two variants where grading differed most between ABC step A and ACMG were the population frequent *CHEK2*(NM_001005735.2) c.599T > C, p.(Ile200Thr) variant (case 1, corresponds to Ile157Thr in other transcripts) and *ABCA4*(NM_000350.3) c.5603A > T, p.(Asn1868Ile) variant (case 10). The *ABCA4* Asn1868Ile variant was e.g., a weak VUS in the ACMG system with average grade 2,7, and a LFE in the ABC system with average grade 3,5. This *ABCA4* variant could represent a hypomorphic allele or be in linkage to such an allele. Hypomorphic variants should by default be graded as a 4 (LFE) in ABC step A.

The ABC step B grade correlated, as expected, with the likelihood of variant reporting in ABC step C: 81-95% of the risk factor (RF) alleles were reported, compared to 65-79% of the variant-of-interest (VOI) alleles (Table 1). The likelihood of a variant being reported was similar between the two classification systems with 3 exceptions: The breast cancer-associated *CHEK2* Ile200Thr variant was more likely to be reported after ABC classification (91%) than after ACMG classification (83% when including maybes). The discrepancy for reporting in favor of ABC was even greater for the periodic fever-associated *TNFRSF1A*(NM_001065.4) c.362G > A, p.(Arg121Gln) variant (81% vs 64%) and the Stargardt disease-associated *ABCA4* Arg1868Ile variant (86% vs 67%). In all three cases, the variant finding matched the clinical picture (Table 1).

We also asked if the laboratory found the standardized comment selection in ABC step C useful. These comments can be tailored to the clinical question. Most responders did find them useful (31/33), and many added additional comments (included in the Excel sheet of Supplementary Table 2). This list of standardized comments can be adapted to local needs or preferences, but one laboratory requested them to be established as a universal standard.

Finally, the choice of ACMG criteria were evaluated among the laboratories providing this information (33–36 of 43). The raw data can be found in Supplementary File S3, and criteria selection concordance rates after removing criteria used less than 4 times (<10%) from the calculation, can be found in Table 2. These concordance rates equal the number of laboratories selecting a certain criterium divided by the number of laboratories responding in the given case. The consistency between laboratories was not great, varying from 27% to 72% (average 46%). On average, only 2 criteria (range 1–4) were used by at least half of the laboratories (Table 2). The poor interlaboratory consistency is evident from the case-specific spreadsheets showing the distribution of criteria used in each case (Supplementary File S3). This wide distribution could also reflect inexperience in selecting ACMG criteria (some selections were clearly wrong), lack of time, or the imposed limitation of clinical and molecular information to what was given in the query (Supplementary File S1). However, it also highlights the challenge of variant classification even with the use of fixed standards. In most cases, the variants were classified after evaluation by several individuals from each laboratory, but we did not ask about their experience. It can be assumed, however, that the majority of the laboratories are experienced based on their reputation and standing in their national healthcare systems.

The comments given were also used to amend the ABC system, see Supplementary File 4 for an updated system overview, and the following changes were made: The standard comments have been expanded to include comments for likely pathogenic variants, likely biallelic recessive variants, and one extra comment option for incidental findings. Step B grade 0 now also applies to cases lacking clinical information. Some laboratories found ACMG preceding ABC classification difficult, a possibility suggested in the original ABC system article [8]. In order to resolve this issue, ACMG criteria may be integrated into the ABC system, and a first suggestion on how this can be done, is found in Supplementary File 4. This suggestion does not incorporate nuances in ACMG criterium strength (very strong, strong, moderate, supporting), but is based on more simplistic selection based on the presence or absence of a criterium. Undoubtedly, this can be improved. Following this preliminary suggestion, the variants classified here got appropriate labels (Supplementary File S4). It should also be noted that many laboratories classified a loss-of-function recessive variant as “pathogenic” in ABC step B, while such variants are risk factors (step B grade 2) that only have clinical relevance in case of a (biallelic) second variant (like in case 3) or a clinical match. If likely biallelic variants are found, an appropriate comment can be selected (Supplementary File 4).

## Discussion

The clinical utility of a test result depends on several factors, and here we only consider if a result was reported or not in a clinical setting where the finding was relevant. In nine of the ten cases in our query, the variant in question was clinically relevant, including the fetal case of a *de novo ANK2* deletion (case 6). A weakness of this study is that only one clinically likely irrelevant finding was included (case 8 with Gly268Ser in *PTPN11* and no known clinical match). The importance of clinical relevance for reporting a variant or not can be illustrated by Bayesian calculations. As an example, we have calculated the likelihood of having autosomal recessive Stargardt disease after the laboratory finds only the frequent disease-associated *ABCA4* Asn1868Ile variant and no second variant—in two different scenarios: one with and one without a clinical fit (Table 3). In a true Stargardt case, the finding and reporting of only this missense variant moderately decreases the likelihood of true disease (likelihood diminishes from a priori ~95% to a posteriori ~83%). In a patient with a Stargardt-unrelated eye condition (like oculocutaneous albinism), the likelihood for having (overlooked) Stargardt disease remains very low (>99%) also after Asn1868ile variant reporting. In this case, the variant should not be reported since the likelihood that the finding is random is 26 times higher, about 8% (gnomAD and reference [10]). More interesting, however, is the clinical consequence of choosing *not to* report this variant as the only finding even if the clinical picture fits with Stargardt disease, i.e., following consensus reporting guidelines in the Netherlands. This may mislead the clinician. The reason is that the likelihood for true Stargardt disease is reduced from a priori 95% to a posteriori 67% if no *ABCA4* variants are reported—even though the common Asn1868Ile variant was found, i.e., a likelihood reduction that is at least twice as large as after variant reporting (95% to 83%). Accordingly, the reporting of low penetrant hypomorphic/disease-associated variants should depend on the clinical question and phenotype. The standardized variant comments of the ABC system are helpful to achieve this, and they also provide other types of flexibility (Supplementary File 4), which is probably also why 31/33 laboratories found such comments helpful.

**Table 3:** Bayesian likelihood for Stargardt disease.

Presence of juvenile macula dystrophy	TRUE	FALSE	NOT REPORTED
Prior probability for Stargardt disease:			TRUE/FALSE
Case A: Clinical picture fits with Stargardt disease in a man 40 years	0.95	0.05	0.95/0.05
Case B: Other cause found for reduced vision in man 40 years	0.01	0.99	
Conditional probability^a^ for Stargardt disease:			
1. An *ABCA4* hypomorphic Asn1868Ile variant detected	0.10*	0.08**	
2. A 2nd *ABCA4* loss-of-function variant NOT detected	0.20*	0.99	
3. No ABCA4 variant reported (despite finding Asn1868Ile)			0.10*/0.91
Joint probability:			
Case A	0.019	0.004	0.095/0.046
Case B	0.0002	0.0784	
Posterior probability:			
Case A: 0.0190/(0.0190 + 0.0040)	0.826	0.174	
Case B: 0.0002/(0.0002 + 0.0784)	0.003	0.997	
Case if no report of Asn1868Ile (0.095/(0.095 + 0.046)			0.67

^a^Conditional probability data based on *Zernant et al. (see ref.’s) and **gnomAD (2 × minor allele frequency).

Another element indirectly related to clinical utility that was not investigated in this study but still warrants discussion, is variant labeling. It is important that genetic test results are not misunderstood because the variant label is misunderstood. The term “pathogenic” means disease causing, and in our opinion this term should not be used for variants that are unlikely to have clinical importance for the individual tested. An example is heterozygosity (carrier status) of a recessive loss-of-function variant in an individual with no symptoms and signs compatible with the recessive disease. Such variants are only pathogenic if combined with a loss-of/reduced function variant in the other allele, but they can be suggestive of a diagnosis if there is a clinical fit (like in the example shown in Table 3). There is also a tendency to use ACMG classification in the VUS–LP–P range to distinguish between low- and high-penetrant variants, and in our study many low-penetrant variants were misclassified as a VUS (Table 1). The ACMG-AMP system was not designed to handle low-penetrant or population-frequent variants, an issue now being addressed by ClinGen’s Low Penetrance/Risk Allele Working Group [6, 11]. One example is heterozygosity of the *F5* Arg506Gln “Leiden mutation” that does not rise above VUS level after ACMG-based classification due to high population frequency. Reporting this variant is clinically relevant in a patient with venous thrombosis, otherwise usually not [12]. Of note, population frequency was not an issue in ClinGen’s recent recommendations concerning established/likely/uncertain risk alleles found after association studies [6]. For these reasons we believe it is better to classify variants based on the consequences they have for gene function (unknown/HFE/LFE/FE) rather than to do it in clinical terms with words like “unknown significance” or “likely pathogenic”. Furthermore, an individual reading his/her medical records may indeed have problems understanding why a pathogenic variant is of no clinical significance.

The functional grading in step A of the ABC system can also easily be aided by bioinformatic tools (like REVEL, missenseAI, spliceAI or paralog comparisons) [13], and there is no need to default the likelihood of variant pathogenicity (to 10%) or the relationship between criteria strengths (as the square root of the value above: 350–18,7–4,3–2,08), as in the Bayesian-based ACMG tool [2]. Similarly, the clinical grading in step B can be aided by tools using HPO terms to prioritize variants, see e.g., the rare disease diagnostic tools (Exomiser) of the Genomics England 100 K study [14].

Finally, our results suggest that elaborating the ACMG criteria even further, as planned, may not increase precision, as intended, because the selection of criteria in challenging cases (like the ones chosen for this study) appears to be quite subjective (Table 2 and Supplementary File S3), and in clear-cut cases there is little need for additional criteria (see Table 2, where the degree of criteria consensus diminish with clinical complexity). However, the integration of molecular rules or AI-based tools into the classification system, as suggested for loss-of-function [15], splice [7] and missense [16] variants in relation to ACMG classification, is a good idea that can also be most helpful for in the functional step A of the ABC system. Another suggestion has been to add “predisposing” and “likely predisposing” as two new ACMG categories [17], but the basic problem is that one-dimensional ACMG classification will always struggle to categorize the spectrum of causes leading to mendelian conditions into a likelihood-of-pathogenicity framework [18]. ACMG classification is nevertheless often very useful because of the systematic approach to variant classification, and ACMG criteria can also be integrated into the ABC system, e.g., as exemplified in Supplementary File S4.

Importantly, the ACMG system should not be used as “proof” for a variant being disease-causing or not, or for deciding when a variant is so likely to be pathogenic that prenatal diagnostics should be offered. This was never the intention. However, according to our personal experience this is how ACMG classification is misunderstood and applied in many places. It should be properly understood, by clinical laboratory geneticists as well as physicians, that the clinical reality is not determined by a variant classification system, but by the patient and/or family. Clinicians must also understand that the laboratory needs clinical information, sometimes even detailed information, to interpret test results. If no clinical information is given, including the reason for requesting the test, the ABC step B grade is 0 and only a functional step A grade can be reported, if desired. Preferably, a laboratory report should be adapted to the clinical question, with highly penetrant dominant or biallelic recessive variants for severe Mendelian disorders as notable exceptions. When reports concern large gene panels that can be requested for highly variable phenotypes, consulting a clinical expert to properly understand the clinical question may greatly improve the quality of the report. The ABC system is well suited for such interaction between the laboratory and the clinic, where the question is more about clinical relevance of the finding(s) than pathogenicity.

### Supplementary information

Supplementary file S1

Supplementary file S2

Supplementary file S3

Supplementary file S4

## Data Availability

All data and a pptx presentation of the ABC system can be found under the News section on the website of the European Society of Human Genetics, www.eshg.org.
