# Supplementary material for: Comparison of the ABC and ACMG systems for variant classification
Source: Eur J Hum Genet. 2024 May 22;32(7):858–63. doi: 10.1038/s41431-024-01617-8 (PMC11219933; doi:10.1038/s41431-024-01617-8)
Supplement: Supplementary file 1 — Supplementary file S1 [file 41431_2024_1617_MOESM1_ESM.docx]

**Supplementary File S1: Invitation letter

Please return to:** [gunnar.houge@helse-bergen.no](mailto:gunnar.houge@helse-bergen.no) within 15.05.2023
_________________________________________________________________________________

Dear variant interpreter(s)

You are invited to participate in a study comparing ACMG and ABC based variant classification, and to explore the usefulness of the standard variant comments of the ABC system. The ABC system was developed by a task force established during my presidency of European Society of Human Genetics.

Your contribution to this study is highly appreciated and will be recognized in a publication with one author per department/center (mark with *) and the other contributors in acknowledgements.

Ten variant cases are presented with the molecular and clinical information that should be the basis of your variant classification. Extensive work, apart from evaluating the variant in the computer system of your preference, should not be done. The classification should thus be based on the information at hand, not on all types of information that may be generated.

The time it takes to do this should be less than an hour, depending on your experience. If possible, you should follow your standard variant classification system, e.g. involving only CLG/molecular biologist(s) or also medical doctor(s).

Best regards

Gunnar D Houge MD PhD
Senior consultant/professor/ESHG treasurer
Department of medical genetics, Haukeland University Hospital, Bergen, Norway
E-mail: [gunnar.douzgos.houge@helse-bergen.no](mailto:gunnar.douzgos.houge@helse-bergen.no)

Name of CLG/molecular biologist(s)

Name of medical doctor(s)

Laboratory/department:

Contact e-mail (person that should be co-author):

Did you find ABC step C useful?

General comments (if any):

**Comparison of ACMG and ABC-based classification, and the value of ABC step C**

Ten challenging cases are picked from our diagnostic routine to
a) compare ACMG- and ABC-based variant classifications
b) examine the consistency of functional (A) and clinical (B) type ABC classification
c) explore if a list of standard variant comments (ABC step C) is useful

ACMG-based classification can be done manually or using a computer-based tool, e.g. <https://www.medschool.umaryland.edu/genetic_variant_interpretation_tool1.html/>

The criteria for ACMG classification would also be appreciated (eg. PS3, BA1, PP4 etc) – but such inclusion is not compulsory.

ABC-classification is based on the system illustrated in the attached .pptx file and the 2021 publication in the Eur J Hum Genet (Open Access, see <https://www.nature.com/articles/s41431-021-00903-z>). Information is also available at the ESHG web site: <https://www.eshg.org/news-home/stepwise-abc-system-variant-classification-of-any-type-of-genetic-variant>

**The ACMG classes are:**

P Pathogenic (5)
LP Likely Pathogenic (4)
VUS Variant of Unknown Significance (3)
LB Likely Benign (2)
B Benign (1)

This classification system do not contain recommendations for standard variant comments.

**The ABC classes are A + B (each ranging from 0-5) resulting in a grade from A to F that forms the basis for step C: the choice of a standard variant comment:**

A – Functional grading

5 Certain functional effect

4 Likely functional effect - *OR* - Hypomorphic allele

3 Variant of unknown significance (VUS) with hypothetical functional effect

2 Likely normal function

1 Normal function

0 VUS that cannot be classified due to lack of information

B – Clinical grading

0 No conceivable genotype-phenotype match

1 Potential genotype-phenotype match (“right type of gene”)

2 Risk factor / a finding that increases the susceptibility for the phenotype

3 Pathogenic, unknown or low penetrance

4 Pathogenic, moderate penetrance (when known)

5 Pathogenic, high penetrance (when known)

**A+B** combined grade (range 0-10): **0-2 = 0
3 = F
4-5 = E
6-7 = D**

**8 = C
9 = B
10 = A**

C – Standard variant comment based on the combined grade from A to F seen above:

**Grade Comment#**

R1437QT915MANK2

We would like to know your choice of variant comment, and please comment on page 1 if you find this option useful or not.

You should do your classification based on the information given below (e.g. you are not expected to further investigate the literature or do structural modelling etc.) – just pretend that this is all the information that it is possible to get. You can of course also check the gene/variant in OMIM and whatever computer program you usually use (like Alamut).

| Case # | Molecular and clinical information given | ACMG | ACMG criteria | ABC - A functonal grade | ABC - B clinical grade |
| --- | --- | --- | --- | --- | --- |
| 1 | *CHEK2*(NM_001005735.2) c.599T>C, p.(Ile200Thr) **Monoallelic variant** gnomAD MAF 0.49% (n=1391), pLI = 0 ClinVar: ~20x LP/P, 10x VUS  CADD Phred: 23,5  Functional assay (good lab): LoF allele  Literature: Numerous articles mentioning the variant as cancer associated  Clinical information given: Female with breast cancer age 41, maternal aunt breast cancer age 38, paternal sister breast cancer age 36. No other finding upon extensive testing (gene panel + MLPA).  **Which standard ABC-comment (from 1-11 above) would you have picked?**  Comment # …………………….  **Would you have reported the variant after ACMG-based classification only?**  YES NO MAYBE | P (5) LP (4) VUS (3) LB (2) B (1) |  | 5  4  3  2  1  0 | 0  1  2  3  4  5 |
| 2 | *CACNA1A*(NM_023035.3) c.4310G>A, p.(Arg1437Gln) **Monoallelic variant**  Variant in extracellular part of Ca-channel between S5 and S6 in domain III. gnomAD MAF 0, pLI = 1  ClinVar: VUS x 1  CADD Phred: 27,8  Functional assays: None  Literature: Found in mother and two children with hemiplegic migraine (PMID 26675662)  Clinical information given: Male 56 with episodic ataxia and dysarthria from age 11 years, nystagmus upon upward gaze and mild ataxia.  **Which standard ABC-comment (from 1-11 above) would you have picked?**  Comment # …………………….  **Would you have reported the variant after ACMG-based classification only?**  YES NO MAYBE | P (5) LP (4) VUS (3) LB (2) B (1) |  | 5  4  3  2  1  0 | 0  1  2  3  4  5 |
| 3 | *ADAMTS18*(NM_199355.4) c.736C>T, p.(Arg246Ter) **Biallelic variants**  c.1718G>C,p.(Arg573Pro)  1) Arg246Ter: Not in any databases or the literature. Introduces a stop in a conserved exon (no 4 of 23), LoF predicted.  2) Arg573Pro: Not in any databases or the literature. Situated in the dimerization domain where Cys577Trp previously has been reported pathogenic (PMID 23818446). CADD Phred: 29.2, Functional testing: Not done.  Clinical information given: Woman 39 years old with microcornea, micrognathia and scoliosis.  **Which standard ABC-comment(s) (from 1-11 above) would you have picked?**  Comment(s) # …………………….  **Would you have reported the variants after ACMG-based classification only?**  YES NO MAYBE | Arg246Ter P (5) LP (4) VUS (3) LB (2) B (1)  Arg573Pro  P (5) LP (4) VUS (3) LB (2) B (1) |  | Arg246Ter  5  4  3  2  1  0  Arg573Pro  5  4  3  2  1  0 | Arg246Ter  0  1  2  3  4  5  Arg573Pro  0  1  2  3  4  5 |
| 4 | *HUWE1*(NM_031407.7) c.12943G>A, p.(Glu4315Lys) **Monoallelic XL variant**  Variant situated in HECT domain (E3 ligase domain), where most other pathogenic *HUWE1* missense variants also are situated. *HUWE1* is X-linked. gnomAD MAF 0.001% (n=1), pLI = 1  ClinVar: 0  CADD Phred: 32  Functional assays: X-inactivation 100% skewed in maternal grandmother, not informative in mother.  Literature: Not reported  Clinical information given: Variant found in two adult MZ brothers with mild ID, microcephaly (-3 SDS) and mild facial dysmorphism. The variant was inherited from their normal mother and grandmother. In the latter X-inactivation was 100/0, in the mother this test was not informative (due to repeat homozygosity).  **Which standard ABC-comment (from 1-11 above) would you have picked?**  Comment # …………………….  **Would you have reported the variant after ACMG-based classification only?**  YES NO MAYBE | P (5) LP (4) VUS (3) LB (2) B (1) |  | 5  4  3  2  1  0 | 0  1  2  3  4  5 |
| 5 | *COL5A1*(NM_000093.4) c.2744C>T, p.(Thr915Met) **Monoallelic variant**  Missense in the middle of collagen-V alpha-1, not in triple helix region gnomAD MAF 0.004% (n=10), pLI = 1  ClinVar: 1 x VUS (patient reported to have classic EDS)  CADD Phred: 32  Functional assays: None  Literature: Not reported  Clinical information given: Adult woman. Surgeon noted “bad skin quality” with pronounced tendency for atrophic scars and striae. Hypermobile joints, especially finger joints. Father also very hypermobile joints.  **Which standard ABC-comment (from 1-11 above) would you have picked?**  Comment # …………………….  **Would you have reported the variant after ACMG-based classification only?**  YES NO MAYBE | P (5) LP (4) VUS (3) LB (2) B (1) |  | 5  4  3  2  1  0 | 0  1  2  3  4  5 |
| 6 | arr[GRCh37] 4q25q26(113,566,399_114,548,378)x1 dn **Monoallelic variant**  De novo 1 Mb deletion of ankyrin-2; *ANK2* (whole gene, pLI = 1); and the delta subunit of calmodulin kinase-II; *CAMK2D* (C-terminal half, pLI = 0.00). No *ANK2* deletions in DGV or other population databases. Heterozygosity for *ANK2* missense variants is associated with cardiac arrythmias/LQTS type 4. The gene is also highly expressed in the brain (both on the mRNA and protein levels).  Clinical information given: Fetus week 21, normal fetal anatomy on US investigation. CVS taken upon patient request for a copy number array test by a private lab. Only finding of interest was the *de novo* deletion mentioned above.  **Which standard ABC-comment (from 1-11 above) would you have picked?**  Comment # …………………….  **Would you have reported the variant after ACMG-based classification only?**  YES NO MAYBE | P (5) LP (4) VUS (3) LB (2) B (1) |  | 5  4  3  2  1  0 | 0  1  2  3  4  5 |
| 7 | arr[GRCh37] 16p11.2(29,567,296_29,794,518)x3 **Monoallelic variant**  227 kb duplication of the telomeric ˜1/3 of the classical ˜0.6 Mb 16p11.2 deletion/duplication region. The duplication contains four protein coding genes, all with pLI values of 0. Region architecture including DNA looping is said to be important for 16p11.2 locus function (PMID 27240531).  Clinical information given: 2-years old girl with poor eye contact and smallish head (-2 SDS), do not like to play with other children, no language. ASD suspected. Otherwise normal. Normal family history. Parental samples not investigated.  **Which standard ABC-comment (from 1-11 above) would you have picked?**  Comment # …………………….  **Would you have reported the variant after ACMG-based classification only?**  YES NO MAYBE | P (5) LP (4) VUS (3) LB (2) B (1) |  | 5  4  3  2  1  0 | 0  1  2  3  4  5 |
| 8 | *PTPN11*(NM_002834.5) c.802G>A, p.(Gly268Ser) **Monoallelic variant**  Variant in the thyrosine phosphatase domain. gnomAD MAF 0, pLI = 0.99  ClinVar: 9 times as LP/P  CADD Phred: 29.4  Functional testing: Not done  Literature: Reported several times associated with “Noonan syndrome” but clinical details usually lacking, and never reported as *de novo*.  Clinical information given: A young man with rhabdomyolysis (with high CK) after strong physical exercise was tested with a gene panel for myopathies with normal findings, and (by mistake) also a rasopathy-panel that revealed this variant as an incidental finding.  **Which standard ABC-comment (from 1-11 above) would you have picked?**  Comment # …………………….  **Would you have reported the variant after ACMG-based classification only?**  YES NO MAYBE | P (5) LP (4) VUS (3) LB (2) B (1) |  | 5  4  3  2  1  0 | 0  1  2  3  4  5 |
| 9 | *TNFRSF1A*(NM_001065.4) c.362G>A, p.(Arg121Gln) **Monoallelic variant**  TNF alpha receptor gene. Associated with periodic fever. gnomAD MAF 1.29% (n = 3646, 29 homozygous), pLI = 0.99  ClinVar: 4 times B, 6 times VUS, 2 times LP/P  CADD Phred: 11.03  Functional testing: Conflicting, but likely mildly reduced protein function.  Literature: Extensive, conclusion: low-penetrant variant clearly associated with TRAPS (TNFR-associated periodic fever syndrome)  Clinical information given: Boy 10 years with recurrent fever episodes.  **Which standard ABC-comment (from 1-11 above) would you have picked?**  Comment # …………………….  **Would you have reported the variant after ACMG-based classification only?**  YES NO MAYBE | P (5) LP (4) VUS (3) LB (2) B (1) |  | 5  4  3  2  1  0 | 0  1  2  3  4  5 |
| 10 | *ABCA4*(NM_000350.3) c.5603A>T, p.(Asn1868Ile) **Monoallelic variant**  *ABCA4* is the only known causal gene of Stargardt-type macular dystrophy. gnomAD MAF 4.2% (n = 11928, 364 homozygous), pLI = 0  ClinVar: 9 times B/LB, 4 times VUS, 3 times LP  CADD Phred: 22.7  Functional testing: No data Literature: Clear evidence that this is a Stargardt-disease associated hypomorphic allele (PMID 28446513)  Clinical information given: Man 40 years with poor vision and strong clinical suspicion of Stargardt-type macular dystrophy.  **Which standard ABC-comment (from 1-11 above) would you have picked?**  Comment # …………………….  **Would you have reported the variant after ACMG-based classification only?**  YES NO MAYBE | P (5) LP (4) VUS (3) LB (2) B (1) |  | 5  4  3  2  1  0 | 0  1  2  3  4  5 |
