# Supplementary material for: Comparison of the ABC and ACMG systems for variant classification
Source: Eur J Hum Genet. 2024 May 22;32(7):858–63. doi: 10.1038/s41431-024-01617-8 (PMC11219933; doi:10.1038/s41431-024-01617-8)
Supplement: Supplementary file 4 — Supplementary file S4 [file 41431_2024_1617_MOESM4_ESM.docx]

**Supplementary File S4: The ABC system in brief 11.04.2024**

| **Step A: Functional grading  5 – FE – F**unctional **E**ffect: Known LoF or GoF variant  **4 – LFE – L**ikely **F**unctional **E**ffect – or known **H**ypomorphic **A**llele  **3 – HFE –** VUS with **H**ypothetical **F**unctional **E**ffect – or a **de novo** VUS  **0 – VUS** without hypothetical functional effect  **2 – LNF – L**ikely **N**ormal **F**unction |
| --- |
| **1 – NF – N**ormal **F**unction _______________________________________________________________________________________ |

**Step B: Clinical grading

0 – NO MATCH variant,** i.e. the gene is unlikely to be linked to the phenotype – or no clinical information

**1 – VOI** - **v**ariant **o**f potential **i**nterest in a gene that fits the phenotype

**2 – RISK FACTOR** variant for the phenotype (recessive or oligofactorial)

**3 – Dominant pathogenic** variant of unknown or low (e.g. <25% lifetime) penetrance

**4 – Dominant pathogenic** variant of moderate (e.g. 25-50% lifetime) penetrance

**5 – Dominant pathogenic** variant or high (e.g. >50% lifetime) penetrance
______________________________________________________________________________________

**Classification from 0/F to A: Combined A+B grade gives the variant class**

**Class**

**0** Step A = 0-2 (no step B) Not reported – and clinical grading not usually done

**F** Step A + step B = 3 Not reported
**E** Step A + step B = 4-5 VOI (Variant-of-interest) group, reporting optional
**D** Step A + step B = 6-7 RISK FACTOR group, reporting recommended if clinical match

**C** Step A + step B = 8 PATHogenic, unknown or low (e.g. lifetime <25%) penetrance

**B** Step A + step B = 9 PATHogenic, moderate penetrance (e.g. lifetime 25-50%)

**A** Step A + step B = 10 PATHogenic, high penetrance (e.g. lifetime >50%)

**X** Step A + step B = 7-10 Incidental or secondary finding

__________________________________________________________________________________

**Step C: Standard variant comments**

**A grade 0-2 (no step B grading needs to be done)**

NORMAL findings

**A+B grade 3 (class F)**

NORMAL findings – no pathogenic or likely pathogenic variants were detected

**A+B grade 4-5 (class E) and 6-7 (class D)**

NORMAL findings – no pathogenic variants that could be related to the phenotype were detected

NORMAL findings – no pathogenic variants that could explain the phenotype were detected

VOI – A genetic variant of potential interest was detected

VOI – Heterozygosity for a recessive variant of potential interest was detected

VOI – Hemizygosity for a variant of potential interest was detected

VOI – Homozygosity for a variant of potential interest was detected

RISK FACTOR – A variant that increases susceptibility for this phenotype was detected

RISK FACTOR – Heterozygosity for a recessive variant of interest was detected

PATH – Likely compound heterozygosity for recessive pathogenic variants was detected
**A+B grade 8 (class C – pathogenic), 9 (class B – moderate penetrance) and 10 (class A – high penetrance)**

PATH – Homozygosity for a recessive pathogenic variant was detected

PATH – Heterozygosity for a dominant likely pathogenic variant was detected

PATH – Heterozygosity for a dominant pathogenic variant was detected

PATH – Heterozygosity for a dominant pathogenic variant of moderate penetrance was detected

PATH – Heterozygosity for a dominant pathogenic variant of high penetrance was detected
**Incidental/unexpected findings and A+B grade 7-10 (class X)**

IF – A genetic variant unrelated to the clinical question was detected

IF – No obvious match between genotype and phenotype. Further clinical investigations necessary

______________________________________________________________________________________

**Suggestion for integration of ACMG criteria in the ABC system:**

**Step A**

5-FE PVS1 PS1 PS3 1 criterium enough to grade

4-LFE PP1-Strong PM4 PM5 2 criteria or more: upgrade to FE

3-HFE PS2 PS4 PM1 PM2 PM6 PP1 PP2 PP3 PP5 3 criteria or more: upgrade to LFE

0-fVUS not enough data to classify
2-LNF BS1 BS2 BS3 BP1 BP2 BP3 BP4 BP5 BP6 BP7

1-NF BA1

Note: One “pathogenic” ACMG criterium is enough to grade.
Known hypomorphic alleles are by default grade 4 - LFE.

**Step B**

0-cVUS BS4, no clinical match or clinical Information
1-VOI gene fits phenotype

2-RISK FACTOR PM3 PP4 1 criterium is enough
3-PATH known pathogenic (AR or AD)
4-PATH known pathogenic (AD, moderate penetrance)
5-PATH known pathogenic (AD, high penetrance)

______________________________________________________________________________________

**Application of ACMG criteria to the eight SNV cases in this study, as suggested above:**

**Case# Gene ACMG criteriae selected ABC-A based on ACMG ACMG only**

1 CHEK2 PS3 FE VUS

2 CACNA1A PM2 PP1 PP3 LFE VUS

3 ADAMTS18-1 PVS1 PM2 FE LP
3 ADAMTS18-2 PM2 PM3 PP3 HFE VUS

4 HUWE1 PM2 PP3 HFE VUS

5 COL5A1 PP3 HFE VUS

8 PTPN11 PM1 PM2 PP3 PP5 LFE VUS

9 TNFRSF1A PS4 BS1 HFE VUS

10 ABCA4 PS4 PP4 LFE (default) VUS
